# Deconstructing stigmatising narratives: a qualitative analysis of contrast devices in interviews with mothers with a mental illness

**DOI:** 10.1186/s40359-024-01933-0

**Published:** 2024-08-09

**Authors:** Monika Schamschula, Jean Lillian Paul

**Affiliations:** 1 Department of Sociology, University of Innsbruck, Innsbruck, Austria; 2 Department of Psychiatry, Psychotherapy, Psychosomatics, and Medical Psychology, Medical University of Innsbruck, University Hospital for Psychiatry I, Innsbruck, Austria; 3 Mental Health Research Program, The Village, Ludwig Boltzmann Gesellschaft, Innsbruck, Austria

**Keywords:** Mental illness, Stigma, Motherhood, Contrast devices, Positioning, Qualitative interview, Rhetorical discourse analysis

## Abstract

**Background:**

In the context of stigma and mental health research, limited empirical studies examine stigma through the positioning of individuals within interview contexts. This study addresses this gap by investigating the positioning processes in interviews with mothers with a mental illness, with a specific focus on the use of contrast devices as a strategy identified through analysis. By analysing how mothers position themselves through contrast devices and to which discourses they refer, this study provides insights into how stigmatising discourses are evident in the narratives of mothers with a mental illness.

**Methods:**

This study is based on 20 semi-narrative interviews with mothers with a mental illness who participated in the *Village Project* (a pilot project co-created for children of parents with mental illness in Tyrol, Austria). Our analysis focuses on identifying stigmatising discourses related to motherhood and mental illness by examining the use of contrast devices in their accounts.

**Results:**

The analysis shows insights into mothers’ efforts to distance themselves from labels such as ‘bad mother’, ‘not normal/crazy women’ and ‘weak person’. These positions often carry a gendered dimension, with motherhood emerging as a central position. Our study highlights the challenges mothers with a mental illness face in navigating societal norms and expectations related to motherhood during research interviews.

**Conclusion:**

The research contributes to a deeper understanding of mental health stigma in the context of motherhood, emphasising the importance of considering gendered dynamics and societal expectations in mental health research.

**Supplementary Information:**

The online version contains supplementary material available at 10.1186/s40359-024-01933-0.

## Introduction

It has been well established that having a mental illness is connected with stigma [1, 2]. Mental health stigma can manifest in various forms, and its analysis can be approached by examining social attitudes toward mental illness [3, 4], by considering the experiences of family members of people with a mental illness [5, 6], and the experiences of individuals affected [7, 8]. To make the underlying stigmatising attitudes and beliefs visible, and to understand and counter stigma, it is important to analyse the discourses that (re-)produce stigma.

In this paper, we aim to examine mental health stigma in the context of motherhood, on the basis of research interviews with mothers with a mental illness. The mothers who were interviewed took part in a pilot project for children of parents with a mental illness in Tyrol, in Austria. The analysis of this article focuses on ways in which stigmatising discourses in the context of motherhood and a mental illness were evident in the narratives of mothers with a mental illness.

Discourse analysis approaches can help to identify stigmatising narratives and explore how discourses perpetuate stigma in various contexts. Rompe, for example, analyses discourses from magazines about ‘mentally ill people’ and shows the ways in which the press disseminates negative and sensationalist portrayals of mental illness [9]. Similarly, Bowen et al., use corpus linguistics to analyse newspaper articles in which the word *schizophrenia* appeared. Their results indicate that the term is often associated with acts of violence, portraying people with schizophrenia as violent [10]. Baer et al., explore the interaction between media depictions of depression and self-portrayals of individuals with depression, finding that the media often depict depression as the antithesis of the dynamic neoliberal norm of a strong and high-performance individual, which they argue impacts the coping mechanisms of those affected [11].

In addition to the diagnosis of a mental illness, factors such as gender [12] and parenthood [13] are important to consider when addressing mental health stigma [13]. Galasiński’s analysis of interviews with fathers with a mental illness highlights the stigma arising from conflicting discourses about masculinity and mental illness. This study underscores how societal views on gender norms impact mental health stigma [14]. Similarly, Halsa’s study focuses on mothers with a mental illness, showing how discourses regarding ‘good motherhood’ and stigmatised discourses around mental illnesses compel women to engage in identity work. The narrative of the caring, self-sacrificing mother contrasts sharply with the stereotype of the irresponsible, dangerous, and self-centred mentally ill person. Mothers with a mental illness can find themselves trapped between the stigmatising discourse of deviance and the prevailing ideology of intensive mothering, which often leads to ambivalence towards mental health support [15]. Galasiński’s and Halsa’s studies demonstrate the value of discursive approaches in examining identity constructions, as also emphasised in a study by Benwell [16].

Tardy’s study also underscores the complex relationship between motherhood and mental health. She applies Goffman’s concept of the backstage and combines this with Simone de Beauvoir’s idea of the ‘counter-universe’. Tardy is interested in what is talked about regarding health issues and motherhood in this ‘counter-universe’ – and, above all, what is not talked about. The author says mothers avoid certain topics because of strong cultural ideas about what makes a ‘good mother’ [17]. Jaworska’s study on online forums, such as *Mumsnet*, illustrates how mothers use these online platforms to share ‘untellable’ experiences with postnatal depression [18]. Unlike Tardy’s study, this analysis shows how women modify dominant motherhood discourses to break the silence around their stigmatised condition.

Overall, the above studies illustrate, as Zayts-Spence et al., point out, that “discourses are powerful.”

“[They] are the means to talk about mental health, the locale where mental health issues are manifest, the means to seek and offer help, and the ways to offer education and develop interventions. They are also the means to challenge and contest negative ideologies” [19 p3].

The specific literature on mothers mentioned here demonstrates how mental health stigma intersects with discourses on motherhood. The study by Jaworska furthermore shows how discourses of motherhood are transformed to enable talking about a stigmatised condition. Building on these findings, our study examines the relationship between motherhood, mental illness, and stigma.

Although there are already valuable studies examining discourses around motherhood with a mental illness and the associated stigma arising from this entanglement, there are none to date that examine the discourses through analysing contrast mothers use in research interviews. Contrast, as a rhetorical device, serves to emphasise differences by presenting one version of the world in juxtaposition with an explicit or implicit opposite [20]. This technique highlights differences between various elements, thereby shaping meaning and establishing membership in situated encounters [21]. In Latimer’s study, for example, it was shown how clinicians used contrast to compare a child with a ‘normally developed child’, thereby demonstrating their ‘clinical evidence’ throughout the consultation [22]. Roberts and Sarangi illustrate how the use of contrast helps medical professionals to show that their own views differ from other professional opinions [23].

Our analysis aims to examine the discursive knowledge that ‘fills’ [24] these contrasts that mothers use in research interviews. Contrast devices enable individuals to distance themselves from other positions, which, in turn, helps them to protect their position. Likewise, contrasts can also serve to appropriate specific positions [25]. As a means of differentiation, contrast structures may be used to analyse constructions of social deviance or social membership [26], making it a valuable tool for analysing stigmatising narratives within relational contexts. By identifying the discursive knowledge the contrasts embody, our aim is to uncover not only the underlying social norms and discourses relating to motherhood and mental illness, but also to shed light on the pervasive stigma surrounding these issues.

We regard contrast devices as a valuable tool for analysing rather sensitive topics, such as mental health and motherhood, as they might offer a nuanced perspective on these topics. Integrating research on discourses that become visible through contrast devices within research interviews can contribute to a deeper understanding of the stigma that mothers face. Considering the current state of research, it is reasonable to assume that discussions on motherhood will be integrated into contrasts of stigmatising narratives.

In this context, it is important to underscore that our article distinctly separates stigmatising subject positions from “actual subjectivations or modes of subjectivation” [27 p92]. The research question posed is as follows: *Which stigmatising discourses do the mothers reference in the interviews employing contrast devices?* The aim is, therefore, to identify the discourses with which the mothers are familiar, take up, and contrast.

## Theoretical framework

Potter and Wetherell argue that interviews should not simply be interpreted as data collection instruments but as conversational encounters [28]. In this context, the interviews are not seen as objective reports, but as a (performative) process of self-representation [29]. Interviews are therefore, not merely events where social practices are discussed, but also moments of lived social practice in which participants position themselves in a situationally constituted social space [30–33] and in which, as with Goffman, the interlocutor’s ‘face’ is to be maintained [34]. From this perspective, how interviewees and interviewers talk about themselves is influenced by their position within this encounter. This aligns with Harré’s position theory, which states that “[w]hat you are is partly constituted by what roles you have [in conversations]” [35 p12]. In this context, people choose discourses that are available to them regarding their position. Positions “enable and limit what an individual feels capable of doing in any given social interaction” [36 p69].

This notion can be further explained using Althusser’s concept of *interpellation*, which Butler applies to gender. *Interpellation* refers to the process by which individuals are addressed by societal institutions and norms, thereby *becoming* specific subjects. Butler provides the example of medical interpellation: “It’s a girl”, transforming the child from an ‘it’ to a ‘she’ [37 p29]. According to this theory, a child *becomes* a girl by being interpellated as such by others in society. This theory suggests that subjects are constituted through language, which stands in the context of binding conventions [37]. Therefore, our language is “never one’s own” [38 n.p.] and never outside the discourse [39 p169].

Mothers interviewed as part of a research project on children of parents with a mental illness are also subject to these principles. When posed with the initial narrative stimulus, “In your opinion, what are the everyday experiences of people with a mental illness?” the women are ‘interpellated’ as individuals with a mental illness. Furthermore, the setting of the *Village*, aimed at supporting their children, positions these women not just as individuals but as mothers with mental illnesses (data collection, methods, and findings of the evaluation can be found in Bauer et al. [40]). Through this lens, utterances are intricately linked to speaker positions, the specific context, and an archive of discourses.

However, it is important to note that individuals’ positions are dynamic and can also conflict with each other [36]. Respondents can position themselves in ways that diverge from the interviewer’s assumptions and external positions, expressing their subjective relevance [30]. This occurs through strategies such as avoidance, refocusing, and re-categorisation [30]. Contrast devices can also be seen as a strategy for negotiating through discourse generated by the interview topic and questions.

## Methods

The analysis is based on 20 semi-narrative interviews with mothers with a mental illness. Semi-narrative interviews allow for asking multiple questions, while still enabling narratives without generating yes-no answers. The flexible interview structure enabled the participants to address topics of interest to us, while also in a way that more likely made sense for them and their lived experiences.

The interviews were conducted during a research project, the *Village Project*, from 2020 to 2022. The *Village Project* is a pilot project in Tirol, Austria, co-developed for children of parents with a mental illness [41, 42]. Each family was recruited by adult mental health practitioners, and families were involved in the project for approximately six months. Participants were screened for eligibility by their treating adult mental health specialists, including assessing the participant’s ability to provide informed consent. Only those deemed capable of providing such consent were referred and their informed consent was overseen by the responsible medical specialist at each referring site. The interviews with the parents were conducted after six months of participation in the *Village Project*, an intervention to enhance (in)formal support around the family and child. The interviews were optional and not a prerequisite for participation in the project. Before the interviews, the women were given an informed consent form to sign. The women were also informed that they could stop the interview at any point, take breaks, and to not answer questions as they preferred.

The interviews were recorded, transcribed, pseudonymised, and translated into English. On average, the interviews lasted for one and a half hours. The Ethics Committee of the Medical University of Innsbruck approved the interview guide (Approval No. ESC 1197/2019). All interviewed mothers (*n* = 20) had at least one child over the age of two living with them, were over 18 years old themselves, lived in Tyrol (Austria), spoke German proficiently, and participated in the *Village Project*. The sample provides some heterogeneity regarding diagnoses, job situations, and level of education (see Table 1).

**Table 1:** Overview of participant features as described by women themselves (*n* = 20)

Participant age	Employment situation	Highest level of education	Type of mental illness (most women described having more than one diagnosis)
Range 24–46 years Mean 37 years	Self-employed *n* = 1 Part-time employment *n* = 10 Unemployed *n* = 4 Sick leave *n* = 4 Maternity leave *n* = 1	Did not finish high school *n* = 4 Apprenticeship *n* = 4 High school or diploma *n* = 6 University degree, *n* = 4 Not specified *n* = 2	Depression *n* = 11 Post-traumatic stress disorder *n* = 7 Anxiety disorder *n* = 5 Addiction *n* = 3 Borderline personality disorder *n* = 3 Dissociative disorder *n* = 2 Obsessive compulsive disorder *n* = 1 Bipolar disorder *n* = 1 Eating disorder *n* = 1 Social phobia *n* = 1 Adjustment disorder *n* = 1

The interview guide contained questions about their experiences during the *Village Project* and general experiences in everyday life (see Additional file 1). After the first narrative stimulus, the order or wording of the further questions was adapted according to the narrative process of the interview, and the conversations were expanded through prompts. We used the interview data to assess the perspectives and encounters of parents participating in the *Village Project* and to delve into children’s and parents’ daily experiences. Within the realm of parents’ daily experiences, one of our interests was stigma, which might influence their ability to access and seek help for themselves and their children.

The transcripts were analysed using thematic analysis by Braun and Clarke [43]. After reading the interviews multiple times, we identified recurring patterns and passages in relation to stigma. Contrast to other people, situations or illnesses emerged as interesting patterns. Based on Goffman’s analysis, which shows that people tend to distance themselves from stigmatised individuals [44], we decided to code the sequences where contrast becomes visible (see Table 2). This coding process, based on both the English and German transcripts, was supported by the QSR International NVivo 12 computer program.

**Table 2:** Codes identified regarding mothers’ use of contrast in relation to stigma and being a mother with a mental illness

Code	Description
to contrast oneself from other mothers (with a mental illness)	emphasising differences from mothers who do not fulfil the ideals of a ‘good mother’ and/or from mothers who are problematic for the child’s welfare
to contrast oneself from others with a mental illness (or from other diagnosis)	comparing one’s own mental health condition with those considered more severe or socially stigmatised
to contrast with healthy or ‘normal’ people or mothers	contrasting with healthy or ‘normal’ people/mothers to explain their mental illness and their situation or the situation of their children
to contrast the past and the current situation	comparing the past and the current situation/self to emphasise that the self or the situation is different

To gain a deeper understanding of the narratives/discourses the mothers contrast themselves to, we used contrast devices as an analytical tool in our discourse analysis. Arribas-Ayllon et al. used the following example from Smith’s study “K is mentally ill” to guide the application [26]:

(i) we would go to the beach or pool on a hot day,
(ii) I would sort of dip in and just lie in the sun,
(iii) While K insisted that she had to swim 30 laps.

In this example, the contrast is created by use of two different activities. Dipping in and lying in the sun is presented as the usual or expected behaviour, while swimming 30 laps is shown as unusual. The pronouns “we”, “I”, and “K” differentiate between the characters. “I” represents the speaker’s behaviour, and “K” represents the contrasting behaviour. The “while” clause highlights the contrast between the two actions simultaneously. The statements are presented as facts, not opinions, which helps establish a baseline of ‘normalcy’ for one behaviour and ‘abnormality’ for the other. Terms like “sort of dip in” and “just lie in the sun” are framed as typical activities, reinforcing what is considered normal [26]. Considering this example, we also see the marked the use of pronouns, linking words or declarations and so on to analyse *how* contrast is introduced into the narratives.

Consequently, we identified the discourses utilised for contrast (such as insisting on swimming 30 laps as not typical behaviour at the beach or pool [26]) in our data. For example, when in our study a mother states *“I still*,* no matter how I feel*,* go out with them every day or to the park; I really do”*, we have analysed in the context of the entire interview, to which narratives the mother contrasts herself [45], and how this narrative segment is ‘produced’ within the encounter, and what is implied by this statement. Furthermore, we identified which stigmatising discourses become recognisable as a result, which involved summarising the stigmatising discursive narratives according to different stigmatising positions.

In the sequence mentioned above, “I” represents the speaker’s behaviour, and “a mother who *does not* go out with her children every day” the contrasting behaviour. The contrast with “a mother who does not go to the park with her children” was generated by an interviewer’s question that focused on behaviour towards children in the context of mental illness. The question about mental illness in the context of parenting prompts the woman to distinguish herself from a mother who does not take her children outside. Subsequently, a discursive relationship between ‘neglecting the children’ and mental illness can become apparent, which the mother uses to distance herself from. We summarised this contrast under the stigmatised position of a ‘bad mother’.

## Results

During our initial research on positioning processes, we discovered that the interviewed mothers use *contrast devices* as a rhetorical strategy to position themselves. In analysing how mothers articulate their experiences with a mental illness and the narratives they employ to distinguish themselves, three key archetypes emerge: the ‘bad mother’, the ‘not normal/crazy woman’ and the ‘weak person’.

Next, we will provide examples from the analysis that demonstrate how women distance themselves from specific narratives, and how these stigmatising positions become evident in this context. We also demonstrate how some positions are interwoven in mothers’ narrations. Above all, the ‘bad mother’ was also identified during the other two emergent narratives. Therefore, the ‘bad mother’ seems to be the most dominant position from which the mothers want to distance themselves within the interviews.

Aside from the negative stereotypes of being a ‘bad mother’, a ‘not normal/crazy woman’ or ‘weak person’, from which they distance themselves, we will also demonstrate how such contrast devices can be identified within text passages that address challenging experiences and concerns about themselves or their children. This highlights how mothers with mental illness, within an interview about their experiences with a mental illness as a parent, must navigate between their identities, experiences, and stigmatising narratives and discourses.

All the examples we used for the analysis were sequences that we had already identified during the coding process. This means that sequences that were not coded – potentially due to displaying more subtle contrasts – are not included in the analysis. We select extracts to demonstrate our analysis and support interpretations. Examples presented are based on two criteria: first, the examples are sequences where mothers make references to themselves (rather than only to their children); second, the examples are sequences where contrasts are used to explain the present situation or the present self (rather than a situation or self from the past).

### Deconstructing the narrative of a *bad mother*

Our analysis shows that the women we interviewed did not share many instances where they were explicitly called ‘bad mothers’ by others. However, they still manifested a strong concern about being perceived as a ‘bad mother’. This was, on the one hand, evident from their narrated fear of being labelled as a ‘bad mother’ and, on the other hand, from their efforts to contrast themselves from negative stereotypes of a ‘bad mother’ during the interview interaction. In the following, sequences are considered in which the ‘bad mother’ becomes the basis for the juxtaposition of the self/other dimension, with particular attention to *how* discursive/rhetorical resources [46] are used to manage one’s own identity as a mother through other-oriented narratives of the ‘mentally ill mother’.

The following excerpt of an interview interaction, for example, is with a mother who first talks about how she feels about a mental illness and how this affects everyday family life. In this context, she explains that she sometimes suddenly needs to go home when she is out with her children because she feels unwell. The interviewer then asks:

*I: And*,* um*,* do you explain to your children then that you have a mental illness or only that you are not well?*

She answers:

*M03: That I’m not doing well. Yea*,* because I don’t want to burden my children too much… I still*,* no matter how I feel*,* go out with them every day*,* or to the park; I really do. When it comes to the children*,* for example*,* I have always*,* I really have to say*,* no matter how I’ve been*,* I’ve always looked after my flat*,* it’s always clean… The children also get everything from me so that// with the children everything is okay. So*,* I’m not one who says < mocking tone: “aargh! the children are too much for me > that I can’t cope”*,* not at all.*

Considering positioning, the focus lies not on the information that the woman goes out with her children daily or that her flat is always clean, but on *how* she brings these statements into the conversation and why they become part of her answer. The fact that the mother (without being asked) adds the information, *“I still*,* no matter how I feel*,* go out with them every day or to the park”*, and *“The children also get everything from me”*, shows how much the mother is confronted with certain ideas of being a mother with a mental illness (whether it is through direct means or indirectly through specific discursive institutions of understanding) and is ‘forced’ to position herself to this idea [15, 17, 47].

In this context, the sequence allows for the interpretation that the interviewed mother is using the statements to contrast her actions with a certain narrative. The woman wants to distance herself from the stigmatising discourse of the mentally ill mother who does not ‘go out regularly with the children’ or who does not manage to ‘keep the flat clean’, etc., by stressing that she does go out *“every day”* and that the flat is clean, and that she is ‘not one of those mothers for whom the children are too much’. In the interview, the mother thus expresses normative expectations of a ‘good mother’ [48] to contrast herself with a ‘bad mother’. This sequence, hence, clearly shows how normative expectations of a ‘good mother’ are introduced during an interview when it is about parenting with a mental illness to distance oneself from certain narratives and ‘supposed’ ways of acting of ‘mentally ill mothers’ and to maintain one’s mother-identity. The emphasis [49] in *“I really do that”*, “*always”*, and *“not at all”* signals how important it is for her to be taken seriously and, in this context, at the same time, implies that she might not be believed (by the interviewer, MS).

The following interview sequence also shows a distinction from ‘bad motherhood’. At the beginning of the interview, the mother reflects on her mental illness with concern for her children’s wellbeing: *“It’s quite difficult. My children*,* for example*,* have been growing up with the fact that I simply have days when I’m not doing so well”* (M01). In this context, the mother’s reflections take place within the discourse that regards raising children of a mother with a mental illness as problematic. Later in the interview, the previous discourse is rechallenged – mainly due to the interview interaction per se:

*I: Do you ever have the fear that your children might somehow come up short*,* if I can put it that way?*

*M01: No*,* because my phases never last long. And*,* um*,* I always look (after)… my children before I look (after) myself. It’s*,* um*,* that my children always come first*,* and even if I’m not doing well*,* I nevertheless try to give everything for them.*

In this section of the interview, the narrative of the ‘bad mother’ is in some way evoked by the interviewer’s questioning of a narrative that the *“children might somehow come up short”*. When asked if this would be the case for her, the mother clearly answers *“no”*. The interviewee revokes the interviewer’s ‘problematic’ request [30] by positioning herself with references to the socially constructed idea of a ‘good mother’ who prioritises her children above all else [50], in contrast to a ‘bad mother’ who fails to meet this ideal.

The term *“nevertheless”* expresses, on the one hand, that her statement needs affirmation, and, on the other hand, how, as a mother, the woman is put in a position – not only by the interview setting but specifically by the interviewer’s question –, in which her actions are permanently evaluated in relation to her mental illness. Even as a mother with a mental illness (this is how the text passage might be interpreted in summary), she *“nevertheless”* does everything to ensure that her children are well. With this statement, it becomes visible that the mother reflects on how others may perceive her as a mother with a mental illness and that she wants to counter this perception.

Several examples in the interviews demonstrate how the mothers use contrast devices when they want to distance themselves from stigmatising discourses of a ‘mentally ill mother’, which is often associated in society with a ‘bad mother’ [15]. Likewise, the examples simultaneously show how the mothers also report the difficulties of having a mental illness as a mother and how, in this context, they, in turn, distinguish themselves in some way from the ideas of a ‘good mother’ or a ‘healthy’ and ‘normal’ person. A sequence that expresses this by using a contrast to ‘normal’ and ‘healthy’ mothers is the following:

*M05: I think there’s a big difference between [people with a mental illness] and people who maybe don’t have a mental illness*,* where it’s not a constant issue*,* can you manage the day? Or can you handle the day with the children? Or*,* um*,* can you cope with the situation in general? Do you think you can cope with this situation? Or is it reasonable for the children? Am I appropriate for the children? When I have the children*,* I simply have to take care of the children; I have to take care of myself*,* which is often a big challenge. Just*,* yes*,* to get through the day without any significant escalations.*

In this sequence, a woman shares her thoughts about being a mother in the context of her mental illness in contrast to ‘people without a mental illness’. This contrast is explicitly drawn by describing her situation in reference to individuals without a mental illness. The mother describes her situation as ‘outside’ the normal [20]. However, M05 – as also in other interviews – not only speaks from the position of a ‘mentally ill mother’ during the interview but at the same time also distances herself from this position. One example:

*M05: Well*,* it’s not that I get up in the morning and can’t motivate myself to get up; I can also take care of the children. […] I’m not this (type of) mother who lies in bed for days*,* because she feels so bad or something*,* and can’t take care of the children at all. I can take care of the children.*

In this sequence, it could be argued that the mother introduces the narrative of the *“mother who lies in bed for days”* to refute it for herself. The rhetorical device of contrast is thus used to negate the discursive narrative of a ‘mentally ill mother’ *“who lies in bed for days”.* The repetition [23] of *“I can take care of the children”* in this sequence emphasises the parenting ability after talking about difficult experiences in daily life.

### Deconstructing the narrative of a *not normal/crazy woman*

In addition to the ‘bad mother’, which is sometimes also introduced as a ‘mentally ill mother’, we also identified the ‘not normal/crazy woman’ as a position from which the mothers contrast themselves. In the following quote, a woman distances herself from a ‘not normal person’. But also in this context, motherhood is immediately brought into the narrative:

*M20: It doesn’t go so far that I have difficulties managing my everyday life. So*,* I can go to work normally; I can take care of the children normally.*

The sequence is an answer to the rather general and open question of which experiences, in their opinion, people with a mental illness have. In the opening interview phrase, the woman reports which experiences she *does not* have as a person with a mental illness. She refers to possible experiences that people with a mental illness may have but which would not apply to her. Specifically, the woman distinguishes herself from people who have *“difficulties [in] managing […] everyday life”* and who cannot *“go to work normally”* and from mothers who cannot *“take care of the children normally”*. So, right at the beginning of the interview, the woman wants to make clear that she is not ‘one of those mentally ill people for whom everyday life, work and childcare do not work’. The term *normal*, used twice in this short sequence, expresses how much a ‘normal life’ is used as a reference point. The fact that she positions herself at the beginning of the interview as someone ‘with this normal life’ expresses that it is not taken for granted that she – as someone who is interviewed in this context – is seen as a ‘normal’ individual. Only after this positioning as someone who can work and care for their children, she talks about the challenges of mental illness.

Moreover, the sequence demonstrates the interwoven narration of leading a normal life and caring for children. The subsequent example also intertwines these themes, illustrating how ‘being a good mother’ and ‘being normal’, or vice versa, seamlessly converge:

*M03: I have*,* for example*,* a neighbour who has four children […]*,* and she really has a full mental illness… and you can really see and hear it with her. I can hear her screaming with the kids from the second to third floors. One time*,* I was passing by her window*,* and I saw her pulling her hair*,* and I thought to myself*,* “What kind of damage is this? […] Well*,* I am nothing compared with this.*

This sequence shows how the interviewed mother introduces another person with a mental illness to position herself as different. Compared to her, the other person has a “*full* *mental illness*”; in this context, the word *“full”* functions as a lexical stress marker to further emphasise the contrast between her and this stereotypical image. Also, the reference to the sense verbs *“see”* and *“hear”* work to further heighten the impression that the neighbour, in a way, embodies a *“full mental illness”*. The person she mentions, however, is not *any* person, but a mother of four children. The portrayal of a stereotypical image of a ‘hysterical mentally ill woman/mother’ implicitly creates a link between motherhood and normalcy.

In addition, it is also interesting that the expression *“damage”* is not only used to describe the neighbour, or the neighbour’s mental condition, but also in other parts of the interview when the mother says that she is afraid that others might think – if they knew that she had a mental illness – that she has *“(mental) damage”* [M03]. How the interviewed woman perceives another woman with a supposed mental illness could be interpreted as simultaneously a fear of how others might perceive her. Similar to this example, the following one also refers to other people with a mental illness:

*M06: And then you are in a department where there are also other patients*,* some of whom are really severe*,* so I still feel normal compared to that*,* um*,* yes*,* and that was really tough to see all the mental illnesses that affect young people […]. There were really people there who were younger than me or the same age or whatever*,* where I thought to myself*,* that doesn’t exist.*

In a story about an inpatient stay at the hospital, a woman brings other patients into the account. With “*I still feel normal compared to them”*, a boundary is made between the *“other patients”* and herself and, simultaneously, a border between her mental illness and ‘serious ones’. The reference to age also implies that her young age is not a determining factor as to why she is in better mental health than the others. With the statements *“that was really tough”* and *“I thought to myself*,* that doesn’t exist”*, she emphasises the severity of what she experienced and, at the same time, reinforces that she is in a different state of health or a different position.

In some other interviews, contrasting from other ‘mentally ill people’ is done by distinguishing between one’s own and other diagnoses. In this context, their own diagnosis is described as ‘more harmless’ or acceptable. One example:

*M13: For me*,* the diagnosis was not as bad as something worse would have been in the end*,* like schizophrenia or borderline [personality disorder]*,* or… deep manic-depressive phases or something like that. So*,* I can live with post-traumatic stress syndrome better and deal with it better than if it had been something else*,* I have to say. Because I can also do something with the diagnosis because I have really had some traumatic experiences that I have not completely worked through.*

In this sequence, the mother seizes on other diagnoses to reflect on her own. According to her, her diagnosis *“was not as bad”*. This is argued by the fact that she *“really had some really traumatic experiences that [she has] not completely worked through”*, i.e., that there is a reason for her psychological condition. Since post-traumatic stress disorder is typically attributed to external factors, such as trauma exposure, individuals diagnosed with this disorder are often socially perceived as bearing less responsibility for their mental health condition compared to those diagnosed with, for example, schizophrenia [51]. Furthermore, it might be the case that stigma-related narratives such as the ‘dangerousness of schizophrenic people’ [52], the ‘laziness or weakness of depressive people’ [53] or the ‘manipulative character of people with borderline personality disorder’ [54] come into play here. The addition of *“I have not completely worked through”* suggests that, unlike the other diagnoses mentioned, post-traumatic stress disorder may be fully processed.

The comparison between her diagnosis and another diagnosis and the subsequent explanation of why her diagnosis is ‘not so bad’ demonstrates, on the one hand, how a need for a cause of the mental illness appears, and how reasons are needed to ‘justify’ having a mental illness or to be able to ‘excuse’ the mental illness in a certain way. On the other hand, it shows the need to differentiate between different mental illnesses and thus normalise her situation. The contrast to other diagnoses is therefore used to present one’s illness as ‘more justified’ and ‘different’.

### **Deconstructing the narrative of a** ***weak person***

The last narrative we identified to which mothers we interviewed contrast themselves is that of a ‘weak person’. This characterisation encompasses traits such as being undisciplined or irresponsible, as illustrated in the following example:

*M04: It is important to go for check-ups*,* to go to the doctor*,* to the psychiatrist*,* and to take medication. Because many say*,* “No*,* I don’t do that*,*” or they stop it again*,* or one day they do and one day not. […] They give up immediately*,* stop it*,* and don’t continue working with the doctor. And it’s important to stick (with) it. So*,* I keep to it regularly. And that’s the be-all and end-all […]. I’ve noticed that a few people give up straight away; they also say something like that// “bullshit*,* that didn’t help” […]. They often annoy me a few when I see that*,* I know a few. Then I think*,* “My goodness*,* pull yourself together”.*

In this sequence, the woman conveys her viewpoint on the behaviours and attitudes of other individuals with a mental illness. Within this context, she incorporates individuals into her account to portray her own identity as someone grappling with a mental illness and to expound on her coping strategies for managing the condition. The transition from the statement *“many say*,* ‘no I don’t do that’*,* or they stop it again”* to *“I keep it regularly”* reveals how she delineates specific patterns of actions and perceptions during the interview, positioning the ‘others’ on one side and herself on the opposite end of the spectrum of actions and perceptions.

The expressions “*it’s important to stick to it”* and *“that’s the be-all and end-all”* signify her belief that her actions are the socially acceptable ‘correct’ ones and that this way of thinking is ‘taken for granted’ and that the interviewer would also share this view [45]. This sequence, as observed in several other parts of the interview, underscores the woman’s intention to distance herself from ‘those with a mental illness who do not seek psychiatric help or take medication’. The statements “*they often annoy me”* and *“my goodness*,* pull yourself together”* underscore her desire to convey in the interview that she cannot comprehend such behaviours and, consequently, seeks to establish a clear distinction from them.

If we look at this sequence in the context of the overall analysis of the interview, we can see that the woman perceives that it is essential to maintain or present agency. ‘Despite’ her mental illness – this is how the analysis can be interpreted – she wants to experience or be seen as a ‘strong, responsible, and disciplined person’ and not be associated with ‘mentally ill people who do not care about their well-being and who do not take responsibility for themselves’. Also, the following example from another mother shows a narrative of a strong person or, more specifically, of a strong mother:

*M01: My psychologist always says I pull myself out of the hole and ensure everything around me functions somehow. Just look less after me*,* but make sure that everything works. And um*,* especially in the phases where I’m just not doing well*,* um*,* I make sure that my children are taken care of*,* that they’re doing well*,* that I get myself together. For today*,* I didn’t feel well at all from early in the morning*,* but I still got up and went to work and*,* in the end*,* somehow got everything together.*

In this passage, the woman calls on the psychologist’s perspective to provide evidence of herself as a strong person. She thus invokes an expert’s opinion, with a higher claim to epistemic knowledge [55] on the topic than her own, thus increasing the strength of her own. This is achieved by constructing the psychologist’s words independently from her as a speaker, which provides a sense of ‘factuality’ [56]. By reporting the psychologist’s evaluation, she emphasises her ability to deal with difficult situations, care for her children, and cope with her everyday life. Therefore, the mother wants to stress how she perseveres through tough times and keeps her life in order, especially while facing personal challenges that distinguish her from other ‘mentally ill mothers’ who may give up in similar situations.

Just as in the pattern of the ‘not normal/crazy woman’, where a reference to ‘bad mothers’ is often made, motherhood seems to be also relevant in the case of the ‘weak person’. With the insertion of “*especially in the phases where I’m just not doing well*,* um*,* I make sure that my children are taken care of*,* that they’re doing well”*, the woman distances herself from the narrative that a mother who is mentally unwell no longer cares for the children. The mental illness may lead to mothers being stereotyped as weak and inadequate caretakers for their children [57]. The concept of the ‘good mother’ who copes without any problems [23] may trigger pressure to position oneself as strong. This sequence reveals the mother’s awareness of potential judgments from others regarding her role as a mother with a mental illness and her intention to avoid such perceptions.

## Discussion

Through the theoretical lens of interviews as conversational encounters [28], wherein participants position themselves within a socially constructed space [30–33], we found that mothers used contrasts during the interviews. Based on the understanding that contrasts allow individuals to distance themselves from certain positions, thereby protecting their stance or acquiring specific roles [25], the use of contrast by mothers in research interviews can be seen as a strategy to distance from negative and stigmatising narratives associated with mental illness or constructing social membership. Therefore, focusing on these contrasts as a rhetorical device [21, 26], we gained insights into how mothers with a mental illness could be seen, how they want to be seen, and how they do not want to be seen.

By analysing the discourses employed for contrast, three primary stigma positions emerged from our data: the ‘bad mother’, the ‘not normal/crazy woman’, and the ‘weak person’ (also described as undisciplined or irresponsible). The ‘bad mother’ introduces narratives that deviate from the idealised norms of a ‘good mother’, portraying her instead as harmful to her children. The ‘not normal/crazy woman’ encompasses the stigma of a woman who is irrational and potentially dangerous, who is unable to manage her daily life. The ‘weak person’ contrasts with the image of a strong, resilient, and disciplined individual. These positions suggest that the stigma associated with mental illness is perceived as akin to individual character flaws, as defined by Goffman [58].

Furthermore, the analysis showed that the respective stigmatising positions were often narratively interwoven. Significantly, the ‘bad mother’ appears as the dominant motif and often overlaps with the ‘not normal/crazy woman’ or ‘weak person’. Both of these stigma positions undermine the role of the emotional cornerstone of the family. Stigmatising narratives in the context of mental illness in mothers, therefore, appear to be closely linked to the role of a mother. This is consistent with studies showing that general parenthood and motherhood can strongly influence stigmatisation processes [12, 13]. The mental health stigma associated with motherhood can be, therefore, different from the experiences of fathers or people without children.

Moreover, our study has shown that contrasts to stigmatising positions can be embedded within narratives about their daily challenges and concerns about themselves and their children. In this context, the mothers link the wellbeing of their children primarily to themselves (and not, for example, to the fathers of the children). Sometimes, these narratives also include comparisons to archetypes of ‘normal people’ or ‘good mothers’, considered healthy and well-adjusted. The presence of different positions which can coexist, clash, and create tensions, as outlined in the literature on positioning [36] becomes evident in the intersection of motherhood and mental illness. The expectation that mothers are the primary caregivers [50, 59–61] may intensify the pressure on women with mental illnesses regarding the societal judgment of their mothering abilities and the accompanying apprehension of being labelled a ‘bad mother’, necessitating efforts to counteract such stereotypes. The simultaneous utilisation of such contrasting elements underscores the conflict-laden nature of women’s accounts, highlighting the intricate interplay between motherhood and mental illness, as already discovered by other studies [15, 47].

However, it also became clear that contrast devices are valuable for talking about experiences with a stigmatised illness. Just as Jaworska [18] highlights that mothers reshape maternity discourses to talk about stigmatised mental illness, our study shows that contrast devices enable mothers to talk about challenges and experiences that could lead to stigma. In this sense, they do not alter the overarching discourse, but rather engage with prevailing narratives surrounding motherhood and mental illness to challenge these discourses for themselves. In this context, it becomes evident that discursive positions can be reflected upon and questioned [30].

Moreover, besides contrast devices, other rhetorical devices were also noticeable. These include, for instance, *emphasis*, *extreme case formulations*, *reported speeches* or *epistemic marking*. This indicates that deconstructing stigmatising narratives should not only concentrate on sequences that utilise contrast devices but also consider other rhetorical devices in the interview material. Our study focused on contrast devices, but examining other rhetorical devices in more depth would be valuable. Furthermore, it might also be worth investigating the anticipated fear of the stigma that parents might have in relation to their children in society.

Finally, we must keep in mind that the sampling was quite specific. Mothers who chose not to participate in the *Village Project* or did not meet the requirements (e.g., due to residence outside Tyrol, Austria, or insufficient knowledge of German) may use contrast devices regarding other positions. It is therefore important to consider the socio-cultural context in which the data collection took place.

## Conclusions

Considering the findings, this study proposes that interviewers should be aware of their role and the context of the interview setting, as well as the stigma associated with the position of the interviewees. Additionally, researchers should consider the dynamics of (stigmatising) positioning processes during the analysis and interpretation of the data. Examining contrast devices within research, therefore, proves beneficial for self-reflection on the researcher’s methodologies and contextualising the research findings. However, the findings could also be useful for other fields, such as psychosocial health, that work with mothers with a mental illness. It can be helpful to consider the stigma positions of the ‘bad mother’, the ‘not normal/crazy woman’, and the ‘weak person’ to create safe spaces for mothers with a mental illness.

### Electronic supplementary material

Below is the link to the electronic supplementary material.

Supplementary Material 1

## Data Availability

The coding manual is available upon request. Raw de-identified data may be made available upon reasonable request from the corresponding author.
