# Supplementary material for: Deconstructing stigmatising narratives: a qualitative analysis of contrast devices in interviews with mothers with a mental illness
Source: BMC Psychol. 2024 Aug 9;12:433. doi: 10.1186/s40359-024-01933-0 (PMC11316300; doi:10.1186/s40359-024-01933-0)
Supplement: Supplementary file 1 — Supplementary Material 1 [file 40359_2024_1933_MOESM1_ESM.docx]

1. **In your opinion, what are everyday experiences of people with a mental illness?** (We are particularly interested in parent’s experiences)
2. **What is your opinion about how people generally perceive individuals with a mental illness?**
3. **What types of support are helpful in challenging situations? Can certain support also be unhelpful or burdensome?**
4. **Have you sought any support when facing challenging situations?** (If yes, what kind of support have you received?)
5. **What motivated you to participate in the *Village* project?**
6. **Before the *Village* project started, how did you envision it to be?** (Was there anything that turned out to be vastly different from how you initially expected it?)
7. **Can you please share your experience with the *Village* project?** (For example, what worked well in the project, what did you experience as supportive, and what would you have liked differently?)
8. **Is there anything in your life that has changed due to the *Village* project?** (Have you noticed any changes in your child?)
9. **As a parent, is there any support you would like to receive?**
10. **Would you recommend the *Village* to families based on your experience with the project?** (Can you explain why (not)? What are your main reasons?)
